# How Arch Support Insoles Help Persons with Flatfoot on Uphill and Downhill Walking

**DOI:** 10.1155/2017/9342789

**Published:** 2017-04-09

**Authors:** Yu-Ping Huang, Kwantae Kim, Chen-Yi Song, Yat-Hon Chen, Hsien-Te Peng

**Affiliations:** ^1^Graduate Institute of Sport Coaching Science, Chinese Culture University, Taipei, Taiwan; ^2^School of Sports Science, Nantong University, Jiangsu, China; ^3^Department of Health Promotion and Gerontological Care, Taipei College of Maritime Technology, Taipei, Taiwan; ^4^Global Action Inc., Taipei, Taiwan; ^5^Department of Physical Education, Chinese Culture University, Taipei, Taiwan

## Abstract

The main purpose of this study was to investigate the effect of arch support insoles on uphill and downhill walking of persons with flatfoot. Sixteen healthy college students with flatfoot were recruited in this study. Their heart rate, peak oxygen uptake (VO_2_), and median frequency (MDF) of surface electromyogram were recorded and analyzed. Nonparametric Wilcoxon signed-rank test was used for statistical analysis. The main results were as follows: (a) peak VO_2_ significantly decreased with arch support insoles compared with flat insoles during uphill and downhill walking (arch support insole versus flat insole: uphill walking, 20.7 ± 3.6 versus 31.6 ± 5.5; downhill walking, 10.9 ± 2.3 versus 16.9 ± 4.2); (b) arch support insoles could reduce the fatigue of the rectus femoris muscle during downhill walking (MDF slope of arch support insole: 0.03 ± 1.17, flat insole: −6.56 ± 23.07); (c) insole hardness would increase not only the physical sensory input but also the fatigue of lower-limb muscles particularly for the rectus femoris muscle (MDF slope of arch support insole: −1.90 ± 1.60, flat insole: −0.83 ± 1.10) in persons with flatfoot during uphill walking. The research results show that arch support insoles could effectively be applied to persons with flatfoot to aid them during uphill and downhill walking.

## 1. Introduction

Uphill and downhill walking exercise is considered a healthy recreational activity. Walking exercise is a popular activity worldwide. “Walking for Health” is the largest organization in England advocating for healthy walking. It consists of 70,000 walkers and encourages more than 63,000 people to engage in regular walking activity. This institution offers more than 3000 short-distance walks per week and provides a solution to the problem of sedentary behavior [1]. Hanson and Jones [2] showed that the benefits of walking exercise include reduction in systolic blood pressure, body mass index, and total cholesterol, among others. Previous studies have proved that walking exercise is beneficial for reducing blood pressure, reducing fasting glucose levels, and increasing VO_2max_ [3]. Moreover, Werner et al. [4] indicated that uphill walking with an inclination of 2% to 8% (equal to 1.2°–4.6°) and constant velocity of each gait on an inclined treadmill can improve the symmetry of the human body. Nowadays, to relieve pressure at the workplace among urban workers, more and more people engage in walking exercise; however, persons with foot issues such as flatfoot are limited in their enjoyment of walking.

Persons with flatfoot have a foot arch support disability in which the midfoot collapses in the medial longitudinal side [5]. The midfoot functions as a shock absorber in the plantar portion, while it allows the foot arch to maintain appropriate elasticity in order to reduce the impact of the ground reaction force (GRF). Constant impact on the plantar aspect by the GRF increases the occurrence of injuries such as heel pain, pelvic malalignment, and plantar fasciitis [6]. Previous studies have identified that functional disorders in the foot could cause lower back, hip, knee, and ankle joint injuries [7, 8]. Persons with flat feet might develop hallux valgus, plantar fasciitis, metatarsal pain, knee and back pain, and other problems without proper treatment [9]. Therefore, persons with flatfoot may not be able to sustain their body weight during prolonged uphill and downhill walking.

In prolonged walking, the arch support is important for mitigating plantar pressure and maintaining dynamic stability [10–13]. Previous studies reported that elderly persons with foot pain or leg symptoms who wore arch support insoles at a minimum duration of 4 h/day for 8 weeks showed improvement in standing balance and prevention of falls [10]. Saadah et al. [13] reported the effect of medial arch support in 16 hospital security guards during standing and walking before and after work and suggested that use of insole support to reduce the foot pressure and muscle work can strengthen the medial arch. Jafarnezhadgero et al. [14] indicated that walking with arch support insoles could reduce the impact of the vertical GRF by 6.9% while increasing the propulsion force by 7% and that the reduction in load rate through the arch support insoles could reduce the risk of lower-extremity injuries and damage caused by the GRF during walking.

Providing a solution, such as the use of arch support insoles, to facilitate participation in prolonged walking exercises and prevent lower-limb injuries is important in persons with flatfoot. Specifically, wearing arch support insoles might return flat feet to the condition of a normal arch. In order to examine the fatigue for people with flatfoot, the current study referred to previous researches which considered the heart rate [15], oxygen consumption [15, 16], and electromyography (EMG) [15, 16] as physical working capacity (PWC) of the fatigue thresholds. The purpose of this study was to investigate the effect of arch support insoles on the heart rate (HR), peak oxygen uptake (VO_2_), and median frequency (MDF) of lower-limb muscles during uphill and downhill walking in persons with flatfoot. We hypothesized that the use of arch support insoles would lower the HR, peak VO_2_, and MDF of lower-limb muscles.

## 2. Materials and Methods

### 2.1. Participants

The participants were 16 healthy college students (age: 18.3 ± 0.7 years, height: 167.5 ± 6.4 cm, weight: 65.1 ± 14.4 kg, body mass index: 23.2 ± 4.7, widest foot width: 8.5 ± 0.7 cm, and narrowest foot width: 6.1 ± 1.1 cm) with flatfoot (defined as an arch index (AI) of 72% ± 10%) [17, 18]. All participants provided written informed consent. This study was approved by the Institutional Review Board of Antai Medical Care Corporation, Antai Tian-Sheng Memorial Hospital (TSMH, approval number 16-107-B1). The inclusion criteria required the absence of lower-limb injuries and of previous surgery in the lower limbs within the span of a year.

### 2.2. Experimental Protocol

Each participant's footprint was recorded with a footprint device (Footdisc Inc., Taipei, Taiwan) during 1 day of the experiment. Then, AI was calculated by using the narrowest foot width divided by the widest foot width from the footprint [17].

Another day was scheduled for the performance of the formal experiment. The participants were instructed to perform 15 min uphill and 15 min downhill walking randomly in standardized footwear (Maximizer16; Mizuno Taiwan Corporation, Taipei, Taiwan) with either a pair of arch support foot insoles (Footdisc) or a pair of flat insoles (Maximizer16, Mizuno Taiwan Corporation). The uphill and downhill walking was a simulated walking on a ±9° [19, 20] inclined treadmill (XG-1812X; New Noble Sport Equipment Co. Ltd., Ningbo, China) with a speed of 0.75 m/s (2.7 km/h) [19, 20]. The hardness of the forefoot, midfoot, and heel of the insoles was measured with a hardness tester (Teclock GS-709N Type A; Teclock Co., Tokyo, Japan).

An HR monitor (H7; Polar Electro Inc., Kempele, Finland) was used to record the HR, and a portable spiroergometer (Metamax 3B; Cortex, Leipzig, Germany) was used to measure the peak VO_2_. Electromyogram (EMG) data were collected by using a Delsys system (Trigno wireless; Delsys Inc., Boston, MA, USA) with a 1000 Hz sampling rate.

The surface EMG (sEMG) sensors were adhered in parallel to the muscle belly of the subject's leg following the direction of muscle fibers. Before adhering the sensors on the muscles, the subjects stood tiptoe to mark the position of the gastrocnemius (GAS), did dorsiflexion to mark the position of the tibialis anterior (TA), did knee flexion to mark the position of the biceps femoris (BF), and did knee extension to mark the position of the rectus femoris (RF) with a black pen [21]. Then, the researchers shaved the participants' skin, removed hair impurities utilizing sandpaper, and cleaned with alcohol cotton sheet in order to gain better EMG signal. Additionally, we fixed the sensors on the leg using breathable tape to ensure the best conductivity and reduce the noise interference.

### 2.3. Data Processing

For dynamic contractions, MDF has been confirmed as a reliable indicator of muscle fatigue [22–24]. EMG spectrum will show lower signal expansion after muscle fatigue [23], and MDF will shift to the left of EMG spectral, that is, the MDF will decrease, which indicates the phenomenon of muscle fatigue [25, 26].

**Figure 1:**
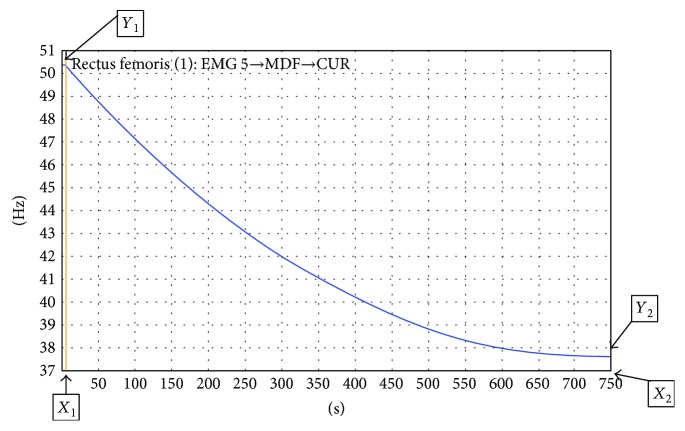
Graph after curve fitting.

The raw data of EMG signals were converted into an MDF-time graph by using EMGWorks Analysis software (Delsys Inc., Boston, MA, USA) with a 0.125 s window length and 0.0124 s overlap. Then, the MDF-time graph was processed by using curve fit calculation (Figure 1). The slope of the curve was calculated to present the decrease/increase of MDF during uphill and downhill walking. The formula of the MDF slope was (*Y*_2_ − *Y*_1_)/(*X*_2_ − *X*_1_) (where *Y*_1_ = first MDF value of the curve, *Y*_2_ = smallest or last MDF value of the curve, *X*_1_ = time of *Y*_1_, and *X*_2_ = time of *Y*_2_).

### 2.4. Statistics

SPSS 18.0 (SPSS Science Inc., Chicago, IL, USA) for Windows was used for statistical calculations. Nonparametric Wilcoxon signed-rank test was used to compare the differences between arch support insole and flat insole in terms of HR, peak VO_2_, and slope of MDF during uphill and downhill walking. The level of significance was set at *p* < 0.05.

## 3. Results

### 3.1. Hardness of Insoles

**Figure 2:**
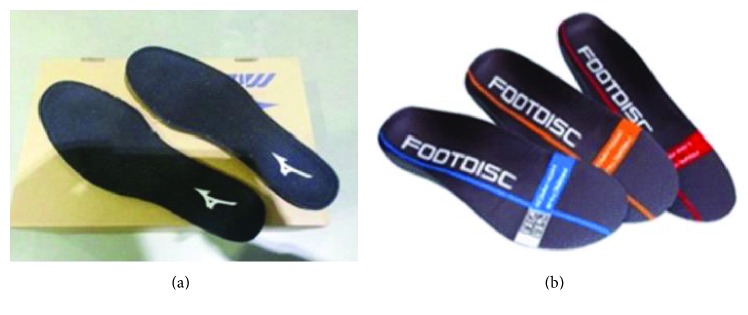
Flat insoles (a) and arch support insoles (b).

The hardness of the forefoot, midsole, and heel areas was measured by using Teclock GS-709N Type A (Teclock Co.) for the two experimental conditions. The flat insole showed 35, 20, and 35 pointers, whereas the arch support insole showed 20, 60, and 20 pointers, respectively (Figure 2) (Table 1). In other words, the material of the arch support insole was harder than that of the flat insole in the midfoot region.

### 3.2. Peak Oxygen Uptake

Table 2 shows the outcomes of parameters during uphill and downhill walking. The peak VO_2_ showed significant differences between arch support insole and flat insole during both uphill and downhill walking (both *p* < 0.001). The peak VO_2_ with arch support insole during both uphill and downhill walking was significantly smaller than that with flat insole based on positive ranks.

### 3.3. Median Frequency

The MDF slope of RF showed significant differences between arch support insole and flat insole during uphill and downhill walking. During uphill walking, the MDF slope of RF with arch support insole (−1.90 ± 1.60 Hz/min) was significantly smaller than that with flat insole (−0.83 ± 1.10 Hz/min) (Table 2) based on positive ranks (*p* = 0.036). During downhill walking, the MDF slope of RF with flat insole (−6.56 ± 23.07 Hz/min) was significantly smaller than that with arch support insole (0.03 ± 1.17 Hz/min) based on negative ranks (*p* = 0.023). No difference was found in the HR and MDF slope of TA, BF, and GAS.

## 4. Discussion

The primary findings of the present study indicated that the peak VO_2_ significantly decreased during both uphill and downhill walking and that the decrease of the MDF of RF was significantly small only during downhill walking with arch support insole.

Wearing arch support insole could be beneficial for persons with flatfoot because their peak VO_2_, which represents the highest value of oxygen uptake in the span of 15 min uphill and 15 min downhill walking, decreased. Hreljac [27] indicated that an increase in exercise intensity, such as from walking to running, would lead to energy expenditure by the plantarflexor and dorsiflexor muscles. Haykowsky et al. [28] indicated that a high intensity of exercise would result in a significant increase of peak VO_2_ compared with moderate-intensity exercise. In other words, peak VO_2_ could be considered the intensity index of body loading. In the aspect of physiology, arch support insoles could reduce the loading of the human body. Therefore, persons with flatfoot who wear arch support insoles may be able to easily engage in the recreational exercise of uphill and downhill walking. We suggest that the arch support insole might effectively reduce the exercise loading due to the impact of uphill and downhill walking.

During downhill walking, RF showed more fatigue with flat insole than that with arch support insole. In previous researches, an effective EMG characteristic analysis for detection of muscle fatigue was based on the MDF, which would be smaller as the muscle fatigue increases [29, 30]. The MDF shift resulted from the change of the conduction velocity [31] and the change in intramuscular pH [32]. In the current study, when participants wore the arch support insole, a significantly lower decrease of MDF was observed only in RF muscle during downhill walking. However, a contrary outcome was found during uphill walking. It could be conjectured to be because of the different contraction types of RF during uphill and downhill walking. The contraction of the RF in uphill walking was considered to be concentric, whereas that in downhill walking was considered eccentric. In general, eccentric contraction was induced by a higher ground impact force compared with that in concentric contraction. Previous researchers indicated that activation of fast-twitch muscle fibers may be associated with a higher risk of injuries in eccentric contraction [33]. The arch support insole for persons with flatfoot could reduce RF fatigue, especially during downhill walking.

During uphill walking, RF showed more fatigue with arch support insole than that with flat insole. This outcome was in contrast to our hypothesis that the arch support insole should cause less muscle fatigue compared with the flat insole during uphill walking because the center of pressure is evenly redistributed on both feet owing to the arch support. Iglesias et al. [34] stated that increasing the insole hardness would increase the physical sensory input. Perry et al. [35] measured different midsole hardness conditions during walking along an 8 m walkway. They found that the range of the center of mass of the whole body increased (soft: 0.14 m, hard: 0.16 m) when the midsole hardness increased. A harder material of insole provides more strength for supporting the leg, which leads to more GRF and increases the range of the center of mass during walking. Yick et al. [36] also indicated that harder insoles would increase muscle activities. In other words, wearing arch support insoles not only could increase the leg support against GRF but also would cause more fatigue of the extremity muscles during uphill walking.

There are two limitations of this study. First, the 15 min uphill and downhill walking exercises were finished within 1 day. Gollhofer et al. [37] indicated that the movement in the conversion between concentric and eccentric contractions during exercise could reduce muscle fatigue; however, the conversion could cause damage to the muscles. The issue may happen to decrease the muscle fatigue during the slope walking in the current study. Second, a flexible flatfoot has an arch support on nonweight bearing but lost the arch support on weight bearing. A rigid flatfoot has loss of the longitudinal arch height [38]. The semirigid flatfoot means not much arch with and without pressure. They all have a common phenomenon which is no arch support on weight bearing. Therefore, in the current study, we recorded the footprint when participants were standing (weight bearing). Uphill and downhill walking are associated with the weight bearing (body weight). Therefore, we did not focus on those impacts of differences in the flexible flatfoot, rigid flatfoot, and semirigid flatfoot.

## 5. Conclusion

Wearing arch support insoles can be beneficial for uphill and downhill walking exercises in persons with flatfoot because the results of this study showed that oxygen uptake was effectively decreased during uphill and downhill walking, and there was less RF muscle fatigue during downhill walking.

## Figures and Tables

**Table 1:** Hardness of insoles.

Areas	Arch support insole (pointer)	Flat insole (pointer)
Forefoot	20	35
Midfoot	60	20
Heel	20	35

**Table 2:** Parameter outcomes during uphill and downhill walking.

	Uphill walking		Downhill walking	
	Arch support insole	Flat insole	Arch support insole	Flat insole
Heart rate (bpm)	141.4 ± 16.0	140.9 ± 14.4	103.6 ± 11.9	105.1 ± 12.6
Peak VO_2_^∗^ (mL min^−1^ kg^−1^)	20.7 ± 3.6^a^^∗^	31.6 ± 5.5^a^^∗^	10.9 ± 2.3^a^^∗^	16.9 ± 4.2^a^^∗^
MDF slope of muscles				
Rectus femoris^∗^ (Hz/min)	−1.90 ± 1.60^a^^∗^	−0.83 ± 1.10^a^^∗^	0.03 ± 1.17^b^^∗^	−6.56 ± 23.07^b^^∗^
Tibialis anterior (Hz/min)	−1.12 ± 1.67	−1.12 ± 1.03	−1.43 ± 1.84	−1.79 ± 2.08
Biceps femoris (Hz/min)	−1.23 ± 1.73	−1.21 ± 0.99	−0.79 ± 1.57	−1.54 ± 0.93
Gastrocnemius (Hz/min)	−1.38 ± 1.63	−1.03 ± 1.25	−1.34 ± 2.25	−2.01 ± 1.72

^∗^Significant difference was found between arch support insole and flat insole, *p* < 0.05. ^a^Based on positive ranks. ^b^Based on negative ranks. *Note*. A negative value of the MDF slope means a decrease of MDF.
